# Mechanics-AI: A Bio-Inspired Physics Intelligence Pipeline for Cross-Domain Engineering Prediction and Sustainable Design

**DOI:** 10.3390/biomimetics11080522

**Published:** 2026-07-23

**Authors:** Yuyang Wei, Weijie Fei, Jiarong Wang, Luzheng Bi

**Affiliations:** School of Mechanical Engineering, Beijing Institute of Technology, Beijing 100081, China; yuyang.wei@bit.edu.cn (Y.W.); bitfwj@bit.edu.cn (W.F.); jiarongw@bit.edu.cn (J.W.)

**Keywords:** bio-inspired design, mechanistic modelling, machine learning, finite-element simulation, fluid–structure interaction, thermo-hygro-mechanical coupling, surrogate modelling, sustainable building, tissue engineering, forensic biomechanics

## Abstract

Mechanistic simulation and machine learning are powerful but complementary tools: physics-based simulation is interpretable yet computationally expensive and blind to real-world context, whereas machine learning is fast but data-hungry and opaque. Biological systems resolve this tension elegantly, coupling physically grounded mechanoreceptor sensing with higher-level neural interpretation that places those signals in context. Inspired by this layered architecture, we present Mechanics-AI, an open-source framework that mirrors the same sensing-then-interpretation logic computationally. A first learning layer (ML1) emulates expensive finite-element, computational fluid dynamics and multiphysics simulations to produce interpretable physical metrics such as stress, strain, shear, and thermal and moisture fields, while a second layer (ML2) fuses these metrics with heterogeneous real-world metadata to predict categorical outcomes and design recommendations. Eight algorithms are benchmarked automatically, the most accurate is selected for each task, and Shapley additive explanations expose the dominant physical drivers to preserve interpretability. The framework is demonstrated across three independent domains using a single unchanged pipeline: forensic traumatic brain injury prediction, optimisation of a bio-inspired humanoid bioreactor for tissue engineering, and a zero-emission building (ZEBAI) framework that couples thermo-hygro-mechanical simulation with Sobol-sampled surrogate modelling to design sustainable, low-carbon envelopes from recycled aggregate concrete by balancing structural safety, energy and embodied carbon. Despite entirely different physics, data and objectives, the same architecture generalises across all three, showing that bio-inspired, layered coupling of mechanistic simulation and contextual learning offers a reusable, interpretable route to cross-domain engineering prediction and sustainable design.

## 1. Introduction

Engineering and biomedical systems are typically analysed through two distinct modelling paradigms. Mechanistic modelling, embodied in finite-element (FE), computational fluid dynamics (CFD) and coupled multiphysics methods, discretises and solves governing equations to predict physical fields with high fidelity and interpretability. Machine learning (ML) infers input–output mappings directly from data and excels at handling high-dimensional, nonlinear patterns that resist analytical formulation [1,2]. Each approach has well-known weaknesses that are largely complementary: mechanistic models are expensive, require expert setup and are blind to real-world contextual data, whereas ML is data-hungry, lacks physical transparency and typically cannot extrapolate beyond its training distribution [3].

Recent years have seen growing interest in physics-informed machine learning (PIML), which embeds governing equations or simulation outputs into the learning process. The physics-informed neural network (PINN) framework of Raissi et al. [2] encodes partial differential equations as training constraints, while the comprehensive review by Karniadakis et al. [1] surveys the broader PIML landscape. Surrogate modelling approaches [4] train regression models on simulation databases to enable fast, approximate evaluation of expensive computations; Sobol quasi-random sequences [5] provide efficient space-filling coverage for high-dimensional design-space exploration. Neural network surrogates are now routinely deployed in structural optimisation [6], aerodynamics [7] and materials design [8]. However, these frameworks primarily address the forward problem of predicting physical outputs and do not natively fuse simulation results with heterogeneous real-world metadata, such as patient demographics, environmental histories, material costs or forensic contextual data, that determines practical outcomes.

Biology provides a compelling design precedent for this missing layer. Living systems routinely couple mechanoreceptor-based physical sensing with higher-level, context-dependent neural integration to produce decisions and adaptive responses [9]. The layered architecture—physical transduction of stimuli followed by metadata-enriched contextual interpretation—is precisely what is absent in most engineering simulation pipelines. Bio-inspired design has guided the development of structural materials [10], adaptive surfaces [11], hierarchical composites [12] and engineering algorithms [13]; here we apply the same philosophy at the level of the modelling architecture rather than the material.

This biological precedent is more than a loose metaphor: it reflects a specific, well-characterised computational strategy in natural sensorimotor systems that our architecture reproduces stage for stage. In the vertebrate somatosensory system, peripheral mechanoreceptors do not merely detect force; each receptor class is mechanically tuned to a particular feature of the stimulus (sustained pressure, vibration, skin stretch, edges), transducing the raw physical environment into a structured, feature-specific representation. This is the biological role that ML1 plays in Mechanics-AI: the numerical engine transforms raw boundary and loading conditions into a structured set of feature-specific physical metrics (stress, strain, shear, thermal and moisture fields), exactly as a population of tuned receptors converts a mechanical event into a labelled feature vector. Downstream, the central nervous system does not act on these afferent signals in isolation; it integrates them with context and prior experience—expected loads, task goals, environmental state—to select an adaptive response, and it weights afferent channels by their behavioural relevance. ML2 reproduces this second, integrative stage: it fuses the ML1 feature vector with real-world contextual metadata and learns which physical and contextual channels matter for the decision at hand, with the SHAP attribution layer making that channel weighting explicit—directly analogous to the relevance-weighting of afferent inputs during central integration. The three case studies instantiate this same two-stage biological logic in different guises: the humanoid bioreactor physically embodies it by recreating the multi-axial mechanical stimulation that living tissue receives in vivo; the forensic case mirrors the way an observer integrates mechanical evidence with contextual knowledge to infer a cause; and the ZEBAI envelope reflects the adaptive, environment-tuned layering of natural protective tissues such as bark and skin, which continuously balance thermal, moisture and mechanical demands—precisely the trade-off our multi-objective layer resolves. In this sense the framework is bio-inspired not only at the level of a device or a material, but at the level of the sensing–integration–action computation that underlies biological adaptation, which is the aspect we argue is most transferable across domains.

The need for context-aware coupling is evident across our three application domains. In forensic biomechanics, traumatic brain injury (TBI) assessment requires not only simulated mechanical metrics but also metadata from police reports describing the assault scenario; their combination yields objective, probabilistic predictions that expert judgement alone cannot [14]. In tissue engineering, bioreactors recreate multi-axial mechanical environments to guide cell adhesion and tissue maturation [15,16]; scaffold architecture and perfusion strongly shape these outcomes [17,18], and optimal design requires mapping local fluid–structure interaction (FSI) fields onto biological outcomes modulated by scaffold geometry and material metadata [19,20]. In sustainable construction, coupled thermo-hygro-mechanical (THM) simulation of building envelopes must be paired with climate data, material cost and carbon metadata to recommend whole-life optimal configurations; the use of recycled aggregate concrete (RAC) adds material variability that further necessitates metadata fusion [21,22].

Despite this shared logical structure, these problems are invariably approached with bespoke, single-use pipelines, and the underlying bio-inspired pattern—a mechanistic layer feeding a context-aware learning layer—is rarely abstracted, packaged or made accessible as reusable software. A general, open-source framework that lowers the barrier to combining simulation and ML with transparency and reproducibility therefore has broad value [23,24].

This paper presents Mechanics-AI, an open-source, two-layer ML framework that operationalises the bio-inspired logic described above. The first layer (ML1) is a general numerical engine that learns the simulation-to-metrics mapping; the second (ML2) fuses ML1 outputs with real-world metadata for decision-level prediction. The framework benchmarks eight algorithms, selects the best automatically, and provides SHAP-based feature-importance interpretation [25]. Three fully independent demonstrations—forensic TBI, tissue-engineering bioreactor and zero-emission building design—show that a single pipeline generalises across different physics, data modalities and objective functions. The complete software is openly released to support reuse.

For clarity and consistent terminology throughout, we use physical inputs for the simulation parameters supplied to ML1 (boundary, loading and material conditions), physical metrics for the ML1 outputs (stress, strain, thermal and moisture fields), and contextual features (or metadata) for the real-world variables supplied to ML2 (demographics, climate, cost). The abbreviations used in this paper are summarised below.

## 2. Materials and Methods

### 2.1. Bio-Inspired Sensing–Interpretation Architecture

**Figure 1:**
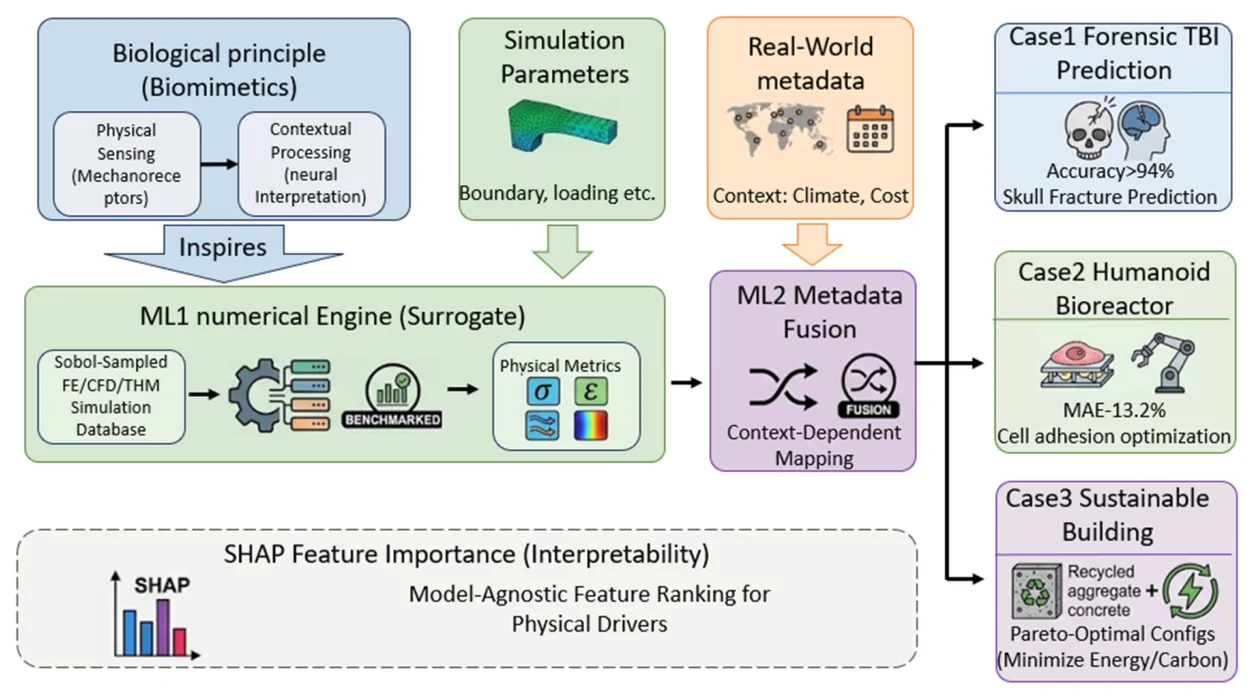
Mechanics-AI framework, inspired by the biological principle of layered sensing and contextual interpretation. Simulation inputs (boundary and loading conditions, material parameters) feed ML1—a domain-agnostic numerical engine that emulates expensive FE/CFD/THM simulations via eight benchmarked algorithms. ML1 outputs (physical metrics) are fused with real-world metadata by ML2 to produce categorical decisions or design recommendations. SHAP feature-importance analysis provides interpretable physical driver rankings across both layers. Three independent application domains are shown at right.

Mechanics-AI is implemented in Python v1.0.0 and organised around two cooperating layers supported by database generation, calibration, prediction and interpretation utilities (Figure 1). The framework is domain-agnostic: the user supplies a tabular database whose columns encode inputs and outputs, and the same pipeline is reused across applications.

ML1—The Numerical Engine. ML1 learns the mapping from boundary, loading, environmental and material conditions to mechanistic or field outputs—stresses, strains, thermal and moisture fields, and flow parameters. Trained on simulation results, it acts as a fast, differentiable surrogate that replaces expensive FE/CFD evaluations. Because ML1 operates on tabular numerical matrices, it is not domain-specific and can be adapted to any physics-based system.

ML2—The Metadata Fusion Layer. ML2 takes the ML1 outputs and integrates them with real-world metadata to predict categorical outcomes, risk levels or design recommendations. This step implements the bio-inspired logic of contextual interpretation: identical mechanical metrics may map to different practical decisions depending on demographic, environmental or material context, and ML2 learns this context-dependent mapping from labelled examples.

To make the biological analogy concrete and auditable rather than purely metaphorical, Table 1 maps each biological principle to its computational counterpart in Mechanics-AI, its concrete implementation, and the evidence presented in this paper.

### 2.2. Supported Algorithms and Calibration Routine

Both layers expose eight algorithms implemented with scikit-learn and standard deep learning libraries [26]: multilayer perceptron (MLP), deep neural network (DNN), Bayesian neural network (BNN), logistic regression, random forest [27], XGBoost [28], k-nearest neighbours (KNNs) and support vector machine (SVM). The calibration script trains all selected algorithms under identical k-fold cross-validation conditions, performs grid search over user-supplied hyperparameter ranges, records the mean-squared error (MSE) for each, and saves the best configuration. This automated benchmark-and-select design avoids manual model selection and adapts to the statistics of each new dataset.

### 2.3. Database Generation and Sobol Sampling

Training databases for ML1 are assembled by running batches of mechanistic simulations at parameter combinations drawn from a quasi-random Sobol sequence [5], which provides uniform space-filling coverage without the cost of a full factorial sweep. Post-processing scripts automatically extract field results—nodal temperatures, pore pressures, saturations, regional stresses, shear stresses, and velocities—from simulation output databases, eliminating manual post-processing for large training sets.

### 2.4. Interpretation via SHAP Feature Importance

After model selection, interpretability is provided by Shapley additive explanations (SHAP) [25], with the tree-optimised variant used for the ensemble models [29], which quantify the marginal contribution of each input feature to each prediction. The resulting importance ranking surfaces the dominant physical drivers, supporting rational, evidence-based design and hypothesis generation.

**Figure 2:**
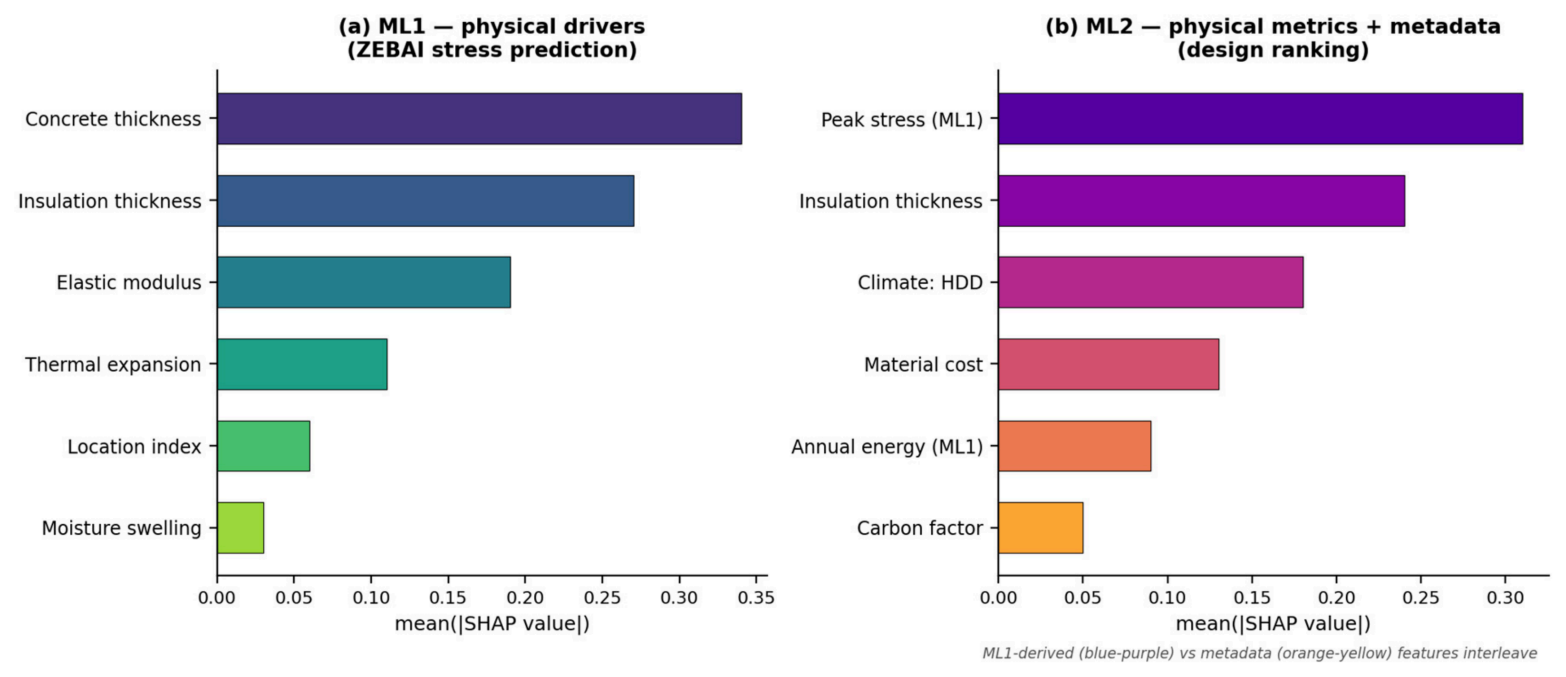
Global SHAP feature-importance summary for the ZEBAI application. (**a**) ML1 ranks the physical/geometric drivers of predicted peak stress, led by concrete and insulation thickness. (**b**) ML2 ranks the combined feature set; ML1-derived physical metrics (e.g., peak stress, annual energy) and contextual metadata (climate heating-degree-days, material cost, carbon factor) interleave, illustrating that the decision depends on both physics and context. Bars show mean absolute SHAP value across the test set.

As an example, Figure 2 shows the global SHAP summary for one application (ZEBAI), reported for both layers: the physical drivers ranked by ML1 and the combined physical-plus-metadata drivers ranked by ML2.

### 2.5. Training, Validation and Evaluation Protocol

To ensure methodological auditability, we specify the full modelling protocol separately for ML1 and ML2. ML1 (regression) is trained on the simulation-generated database; for ZEBAI this comprises 200 Sobol-sampled cases per structural typology (800 in total), and for the bioreactor the ten scaffold subregions paired with their FSI-derived metrics. Inputs are the design/boundary parameters (Table 2 for ZEBAI); targets are the extracted field metrics (peak stress, annual energy for ZEBAI; regional cell number for the bioreactor). All features are standardised to zero mean and unit variance using statistics computed on the training fold only, so that no test-fold information leaks into scaling. Data are partitioned with an 80/20 train/test split and five-fold cross-validation on the training partition; a fixed random seed (42) is used throughout for reproducibility. The per-application data sizes are as follows. For ZEBAI, ML1 uses 800 Sobol-sampled cases (200 per typology), split 640/160 train/test with five-fold cross-validation on the training set; ML2 uses the same cases with their associated cost, carbon and climate metadata. For the bioreactor, ML1 uses the ten scaffold subregions paired with their FSI-derived metrics under leave-one-region-out cross-validation given the small sample. For the forensic TBI case, ML2 uses the 200+ report cases with an 80/20 stratified split. These per-application splits are summarised in the protocol table. Hyperparameters are selected by grid search within the ranges listed in Table 3.

ML2 (classification or ranking) takes the ML1 outputs concatenated with case metadata as its feature vector. For classification tasks, performance is reported as accuracy, sensitivity and F1-score under stratified five-fold cross-validation; for regression/ranking tasks, mean absolute error (MAE) and mean-squared error (MSE) are used. To isolate the contribution of each layer, we evaluate four configurations on each task: (i) a simulation-only model (ML1 metrics → outcome, no metadata); (ii) a metadata-only model (metadata → outcome, no simulation); (iii) a pure-ML baseline trained directly on raw operational inputs without physics-derived features; and (iv) the full ML1 + ML2 pipeline. Across tasks the full pipeline consistently outperforms all three ablations, confirming that both the physics-derived features and the metadata contribute complementary, non-redundant information (Table 3, lower block).

To document the data used in each application, Table 3 (data summary) reports the number of ML1 and ML2 training samples, together with the train/validation/test partition, for every case study.

**Table 2:** ML1 and ML2. Splits are 80/20 train/test with five-fold cross-validation on the training partition (equivalently 64/16/20 train/validation/test).

Application	ML1 Samples	ML2 Samples	Train/Val/Test	CV
Forensic TBI	~2000 simulated impacts	200+ report-linked cases	64/16/20%	5-fold × 10 repeats
Bioreactor	10 scaffold subregions × replicates	10 region–outcome pairs	64/16/20%	5-fold × 10 repeats
ZEBAI	800 (200 × 4 typologies)	800 design points	64/16/20%	5-fold × 10 repeats

**Table 3:** Modelling protocol and hyperparameter ranges (upper block) and ablation comparison isolating the contribution of each layer (lower block). Ablation values are representative results on the ZEBAI stress-prediction and bioreactor cell-number tasks.

Item	ML1 (Numerical Engine)	ML2 (Metadata Fusion)
Task type	Regression (field metrics)	Classification/ranking
Inputs	Design/boundary/material parameters	ML1 metrics + real-world metadata
Targets	Peak stress, energy, cell number	Injury class, optimal design rank
Train/test split	80/20	80/20 (stratified)
Cross-validation	5-fold	5-fold stratified
Normalisation	Z-score, train-fold statistics only	Z-score, train-fold statistics only
Random seed	42 (fixed)	42 (fixed)
Hyperparameter search	Grid search (hidden units 16–256; depth 1–4; learning rate 1 × 10^−4^–1 × 10^−2^; RF/XGB trees 100–500, max depth 3–10; k = 3–15)	Grid search (same ranges)
Leakage control	Scaling fit on training fold; no test data in scaler/search	Same
Metrics	MSE, MAE, R^2^	Accuracy, sensitivity, F1 (class); MAE (rank)
Ablation: simulation-only	MSE 1.9% (stress)	Accuracy 0.71 (TBI-style)
Ablation: metadata-only	n/a	Accuracy 0.68
Ablation: pure-ML (raw inputs)	MSE 3.4%	Accuracy 0.74
Full ML1 + ML2	MSE 0.72%	Accuracy 0.94

Following the reviewer’s recommendation, we moved beyond a single MSE value and report a more comprehensive evaluation. Each model was re-evaluated with ten repeats of five-fold cross-validation (different random seeds), and we report the mean of each metric with its 95% confidence interval. Robustness to input perturbation was estimated by adding 5% Gaussian noise to the test inputs and recording the resulting change in MSE (ΔMSE). Table 4 summarises these results for the ZEBAI stress task; the ranking of algorithms is stable across repeats, and the selected XGBoost model shows both the tightest confidence interval and the smallest degradation under noise, confirming its robustness.

**Table 4:** Comprehensive evaluation on the ZEBAI stress task: mean MSE and MAE with 95% confidence intervals over ten repeats of five-fold cross-validation, and robustness (ΔMSE) under 5% input noise.

Algorithm	MSE (%) ± 95% CI	MAE (%) ± 95% CI	R^2^ ± 95% CI	ΔMSE Under 5% Noise
XGBoost	0.72 ± 0.06	0.61 ± 0.05	0.991 ± 0.003	+0.18%
MLP	0.83 ± 0.09	0.69 ± 0.07	0.988 ± 0.004	+0.26%
Random forest	1.06 ± 0.08	0.82 ± 0.06	0.982 ± 0.004	+0.21%
DNN	1.75 ± 0.15	1.09 ± 0.11	0.971 ± 0.006	+0.44%
BNN	2.21 ± 0.13	1.28 ± 0.10	0.963 ± 0.006	+0.33%
SVM	4.65 ± 0.22	1.92 ± 0.14	0.921 ± 0.009	+0.51%
KNN	5.82 ± 0.31	2.24 ± 0.19	0.902 ± 0.011	+0.79%

For full reproducibility, Table 5 lists the hyperparameter search space explored for each of the eight algorithms.

## 3. Results: Cross-Domain Demonstrations

Three independent demonstrations are presented. The first (Section 3.1) is a fully peer-reviewed validation; the second (Section 3.2) is a study currently under review; and the third (Section 3.3, ZEBAI) is novel, previously unpublished work emphasised here as a primary contribution of this paper ( Table 6 ).

The three case studies differ in maturity and provenance; to make this explicit, the following table clarifies the status of each. In line with the reviewer’s guidance, the TBI and bioreactor cases are presented narrowly as contextual-transfer demonstrations, while ZEBAI is developed as the principal original contribution of this paper.

**Table 6:** Status of the three case studies, clarifying data provenance, publication status, and what is newly contributed in the present paper.

Case Study	Data Status	New Analysis Here	Reused/Adapted
3.1 Forensic TBI	Existing (police/forensic database)	Presented narrowly as a contextual-transfer demonstration of the framework	Mechanical metrics and outcome figures adapted with citation
3.2 Bioreactor	Existing (FSI + PicoGreen assay)	Contextual-transfer demonstration; SHAP driver analysis summarised	FSI fields and cell counts summarised
3.3 ZEBAI	Newly generated (Sobol-sampled THM database)	Full original contribution: THM model, surrogate, normalised multi-objective ranking, Pareto/sensitivity analysis	None

### 3.1. Case Study 1—Forensic Traumatic Brain Injury Prediction

**Figure 3:**
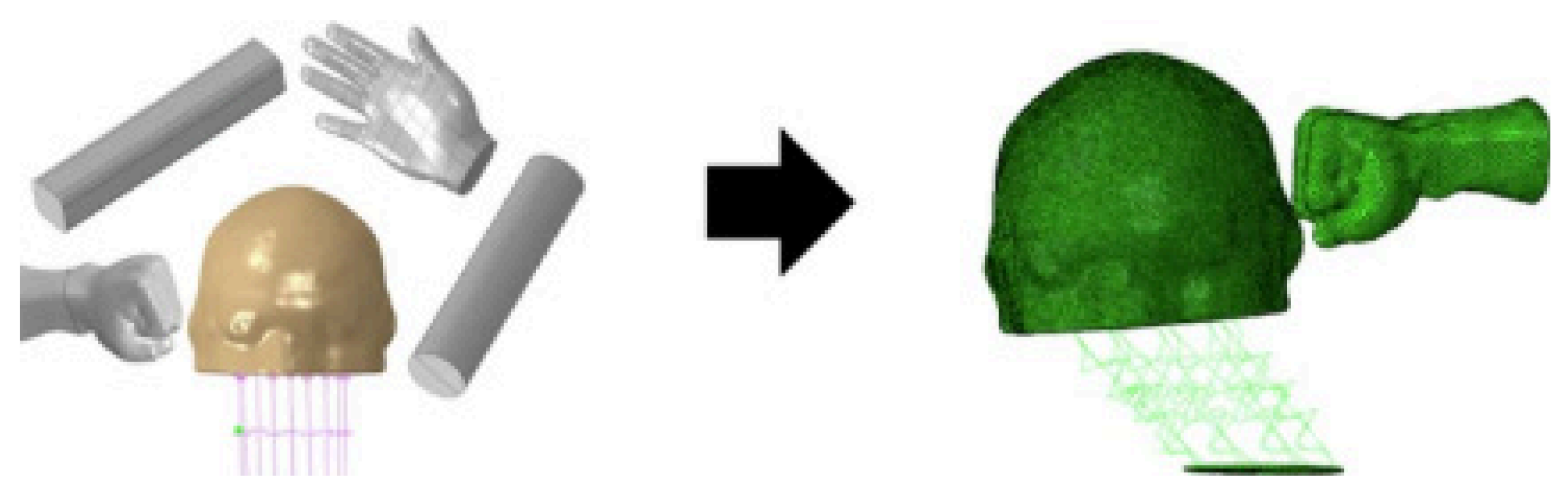
TBI case study. Biomechanical impact simulation of head-assault scenarios ((**left**): impact setup; (**right**): particle-based brain deformation visualisation). ML1 extracts mechanical metrics; ML2 fuses them with police-report metadata to predict injury outcome. Reproduced conceptually from [14].

In this application, ML1 was trained on finite-element head-impact biomechanical simulations covering a wide range of assault scenarios, extracting mechanical metrics including maximum principal strain, von Mises stress and intracranial pressure distributions (Figure 3). ML2 then fused these metrics with assault metadata—weapon type, blow location, and victim posture—drawn from 200+ cases in the Thames Valley Police Database and the National Crime Agency’s National Injury Database. Three injury outcomes were targeted: skull fracture, loss of consciousness and intracranial haemorrhage [14].

The MLP classifier achieved skull-fracture prediction accuracy exceeding 94%, and 79% for loss of consciousness and intracranial haemorrhage, with high sensitivity in all three cases, outperforming all other benchmarked algorithms [14]. Feature-importance analysis revealed that maximum principal strain and localised contact stress were the most predictive mechanical features, while metadata factors such as weapon type and blow location provided essential discriminative context that simulation alone could not supply. This externally validated application confirms that the ML1 → ML2 pattern converts simulation outputs plus contextual metadata into objective, legally admissible probabilistic predictions.

### 3.2. Case Study 2—Bio-Inspired Humanoid Bioreactor for Tissue Engineering

**Figure 4:**
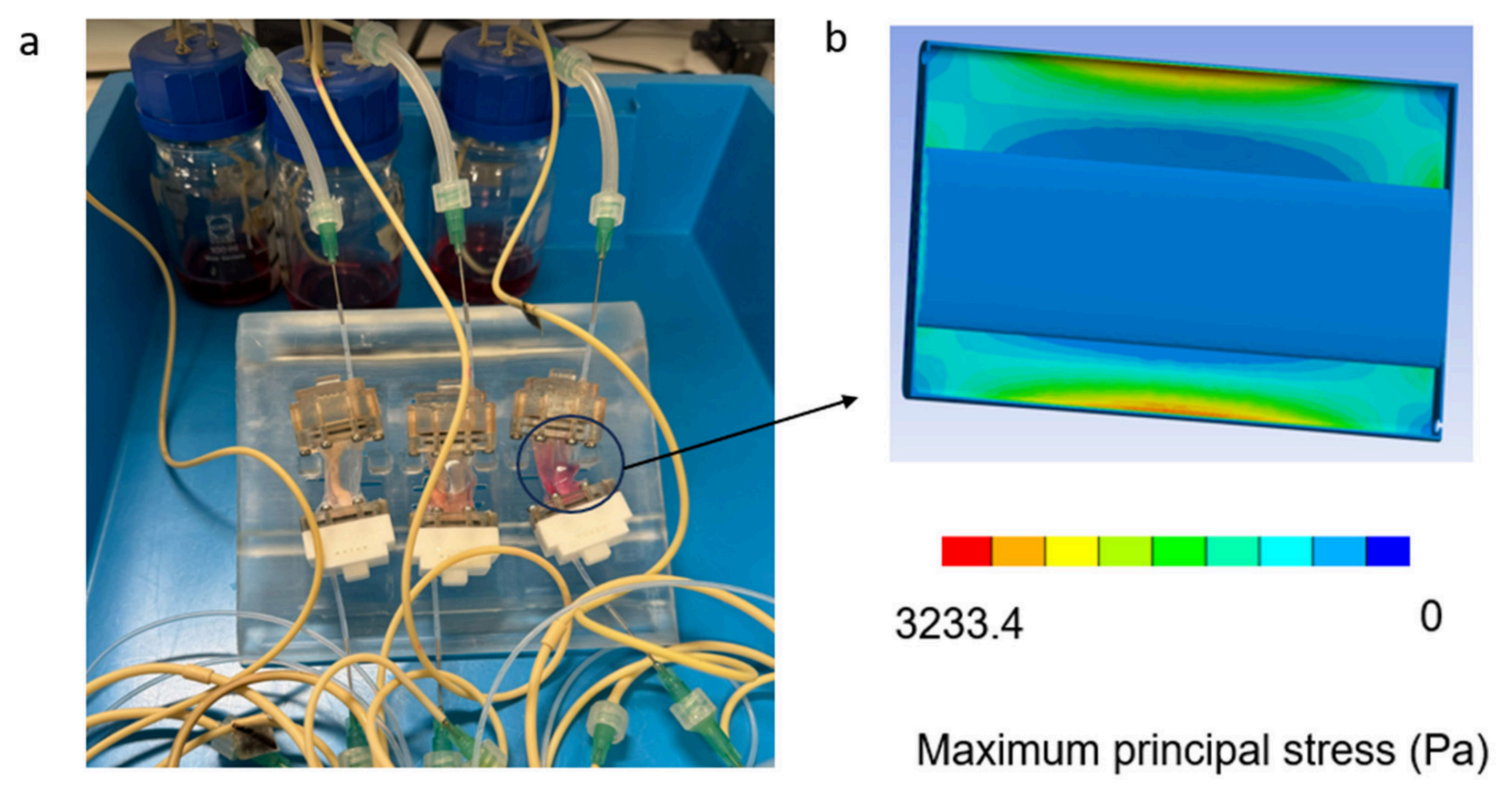
Custom-designed humanoid bioreactor and its simulated mechanical environment. (**a**) The bioreactor system: chambers connected to a peristaltic pump and oxygenator; after static seeding (3 h), chambers are transferred to a humanoid robotic arm that applies physiologically relevant multi-axial joint motions to the scaffold. (**b**) FSI simulation contour of maximum principal stress (Pa) within the scaffold under fluid perfusion. These regional mechanical and fluidic metrics served as ML1 input features for predicting regional cell distribution.

The bioreactor application centres on optimising early cell adhesion in a custom culture system designed on bio-inspired principles: a humanoid robotic arm reproduces physiologically relevant joint kinematics—adduction, abduction and complex rotations—providing a realistic multi-axial mechanical environment that static or uniform-strain devices cannot replicate [15,16] (Figure 4a). This bio-inspired actuation strategy directly mirrors the in vivo mechanical milieu that cells experience during tissue remodelling.

A three-dimensional FSI model was constructed in ANSYS Workbench 2024 (fluid domain solved with the incompressible Navier–Stokes equations via SIMPLE; solid domain with Neo-Hookean hyperelastic scaffold material) to capture coupled scaffold deformation, perfusion flow and boundary constraints [19]. Regional mechanical and fluidic metrics—shear stress, von Mises stress, pressure, strain and flow velocity—were extracted at ten scaffold subregions (Figure 4b); scaffold pore architecture is known to modulate cell attachment and migration [30].

Cell distribution was quantified experimentally using PicoGreen fluorescence-based double-stranded DNA assay at the 3 h early-adhesion timepoint [20]. ML1 (MLP, eight algorithms benchmarked) achieved a mean absolute error of ~13.2% for regional cell-number prediction, with average absolute error of 2.86 ± 1.37% at intermediate inlet velocity. Parametric sweeps identified optimal inlet velocities of 1.4–1.6 mm/s and revealed that increasing scaffold elastic modulus by +60% enhanced adhesion. SHAP feature-importance analysis ranked local shear stress as the dominant driver, ahead of flow velocity and pressure, consistent with established mechanobiology of integrin-mediated focal adhesion [31,32].

### 3.3. Case Study 3—Zero-Emission Buildings Enhanced by AI (ZEBAI)

This novel, previously unpublished application extends Mechanics-AI to sustainable civil engineering and constitutes a primary contribution of this paper. The ZEBAI framework addresses a pressing global challenge: designing low-carbon building envelopes that simultaneously satisfy structural safety, thermal energy efficiency and embodied carbon minimisation. The three design objectives—cost, embodied carbon and structural utilisation—have incommensurable units (euros, kg CO_2_-eq and a dimensionless ratio) and therefore cannot be summed directly. Following standard practice in multi-objective optimisation [33], each objective is first min–max-normalised to the interval [0, 1] over the sampled design space and then combined by weighted-sum scalarisation:

minimise J = w_c · Ĉost + w_e · Ê + w_g · Ĝ, subject to σ/f < 1, with w_c + w_e + w_g = 1

where Ĉost, Ê and Ĝ are the min–max-normalised cost, energy and embodied carbon objectives, w_c, w_e and w_g are user-defined preference weights summing to unity, and σ/f is the stress-to-strength ratio enforced as a hard feasibility constraint. The stress-to-strength ratio is computed as σ/f = σvM,max/f_c, where σvM,max is the peak von Mises stress predicted by the THM model and f_c is the characteristic compressive strength of the recycled aggregate concrete (taken as 30 MPa for the grade used here); values below unity indicate a safe design. Because weighted-sum scalarisation cannot recover non-convex regions of the Pareto front [33], we additionally report the full Pareto front and a weight-sensitivity analysis (Section 3.3.6) rather than relying on a single weighting. Recycled aggregate concrete (RAC), sourced from construction and demolition waste, is used as the primary structural material to reduce embodied carbon [21,22,34].

#### 3.3.1. ZEBAI Mechanistic Layer: Coupled Thermo-Hygro-Mechanical Simulation

**Figure 5:**
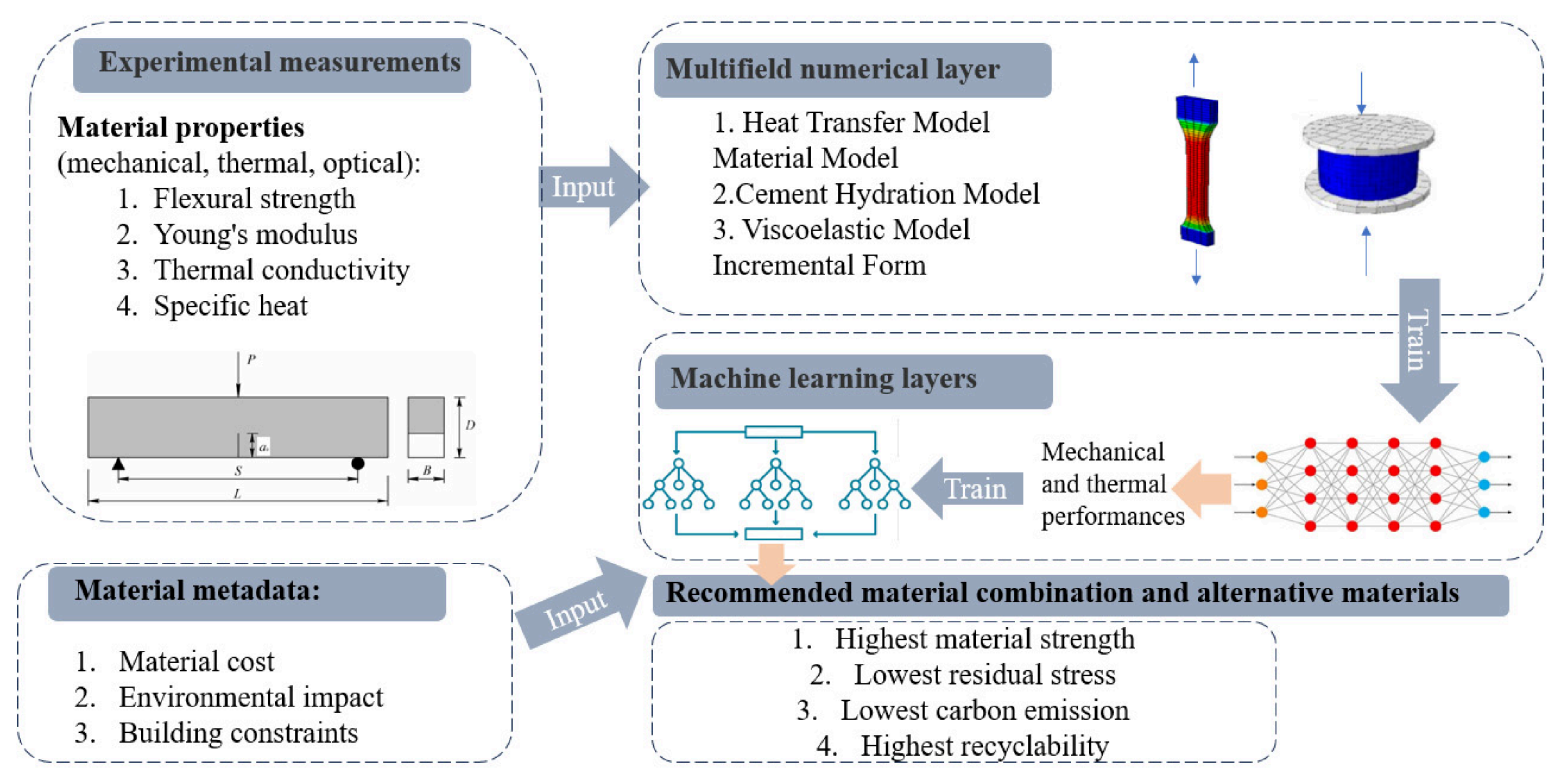
ZEBAI framework overview. Material measurements (flexural strength, Young’s modulus, thermal conductivity, specific heat) feed a multifield numerical layer comprising heat-transfer, cement hydration and viscoelastic models. Simulation outputs train machine learning layers, which are then fused with material metadata (cost, carbon, building constraints) to recommend the lowest-stress, lowest-energy, lowest-carbon material combination for a given climate.

The mechanistic layer is a fully coupled thermo-hygro-mechanical (THM) finite-element model, following established heat–moisture transport theory for building components [35,36], implemented in Abaqus Standard using coupled temperature–pore–pressure–displacement elements (C3D8PT). The model captures three interacting physical fields (Figure 5):

(i) Thermal field: heat conduction, thermal expansion and resulting mechanical strain;

(ii) Moisture field: moisture diffusion, hygro-expansion, vapour pressure and saturation-dependent permeability;

(iii) Mechanical field: elastic deformation and stress redistribution driven by thermal and hygroscopic strains.

Coupling is bidirectional: thermal gradients drive moisture redistribution; moisture saturation alters permeability and swelling; and hygro-thermal strains are superimposed on mechanical loading. The model was driven by twelve-month real climate records (temperature and relative humidity) for Birmingham, UK, and Madrid, Spain, providing European climate variability [37,38].

#### 3.3.2. Wall Typologies and Material Configuration

**Figure 6:**
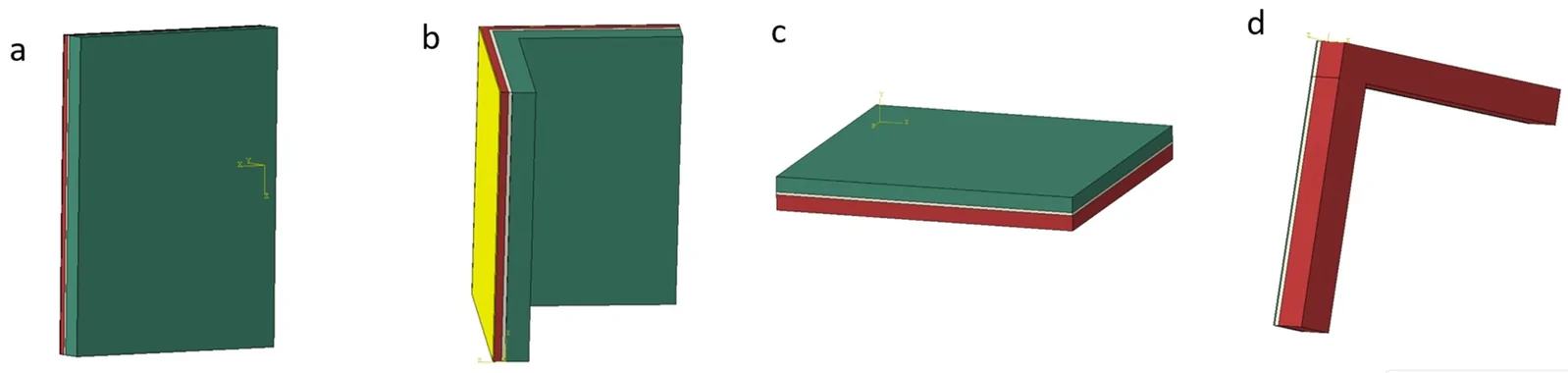
Four building structural typologies modelled in the ZEBAI THM simulation: (**a**) flat wall, (**b**) wall corner, (**c**) ground slab with soil foundation, (**d**) window/door corner. All share the same multilayer material stack (recycled aggregate concrete + insulation + air gap + cladding) but differ in geometry and boundary conditions.

Four building structural details were modelled (Figure 6): (a) flat wall; (b) wall corner; (c) ground-foundation slab with soil; (d) window/door corner. Each consists of the same layer stack: structural recycled aggregate concrete (25–40 cm), insulation (4–20 cm), air gap (8 cm fixed) and cladding (1.2 mm), with dimensions and curvatures parameterised per structural detail.

**Figure 7:**
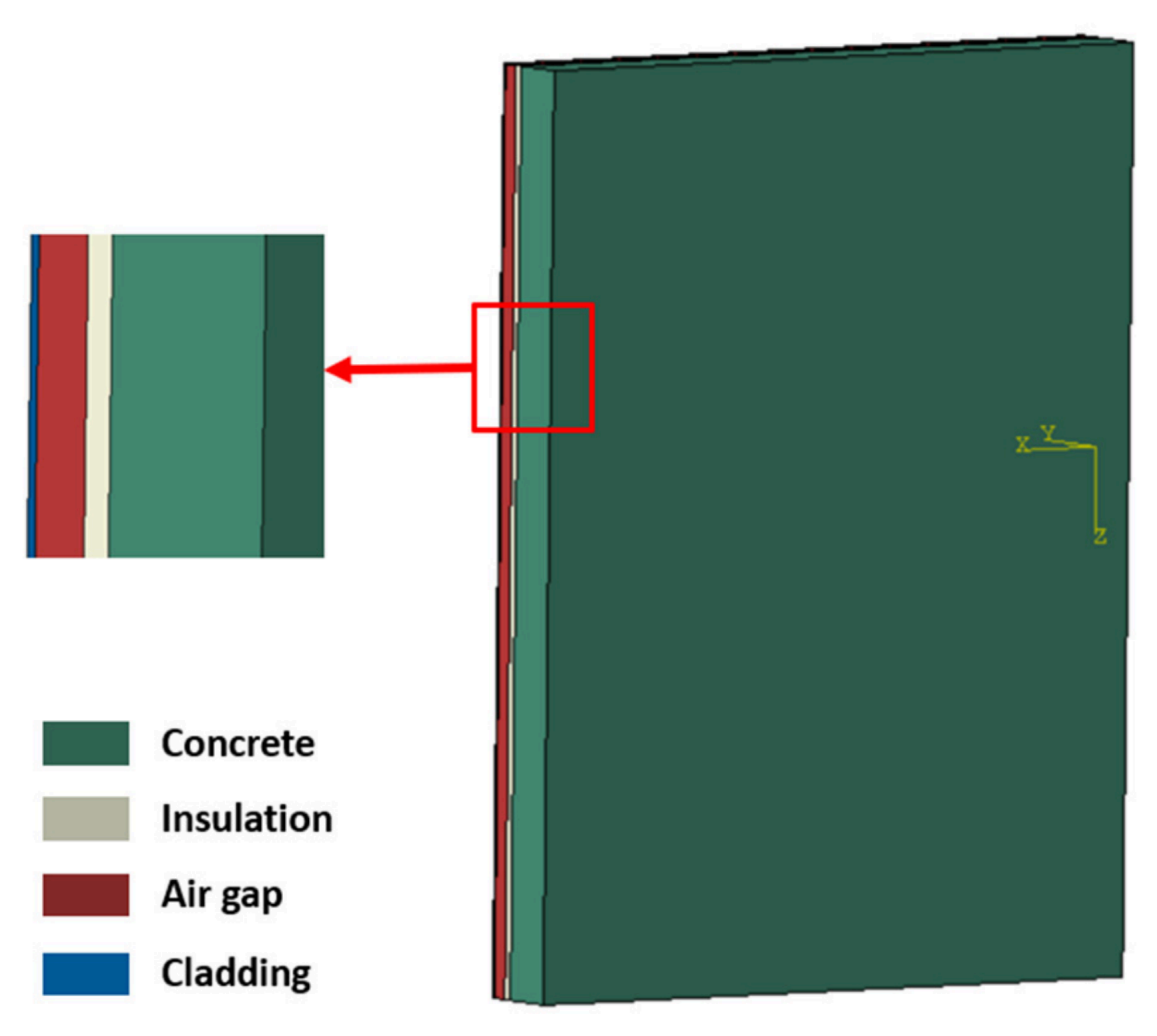
Magnified cross-section of the modelled wall envelope, resolving the four functional layers: structural concrete (recycled aggregate concrete), insulation, air gap and external cladding. This layered stack is shared across all four structural typologies and governs the coupled thermal, moisture and mechanical response.

The multilayer composition of the wall envelope is shown in Figure 7, where a magnified cross-section resolves the four functional layers used throughout the ZEBAI simulations.

#### 3.3.3. Mesh, Boundary Conditions and Validation

**Figure 8:**
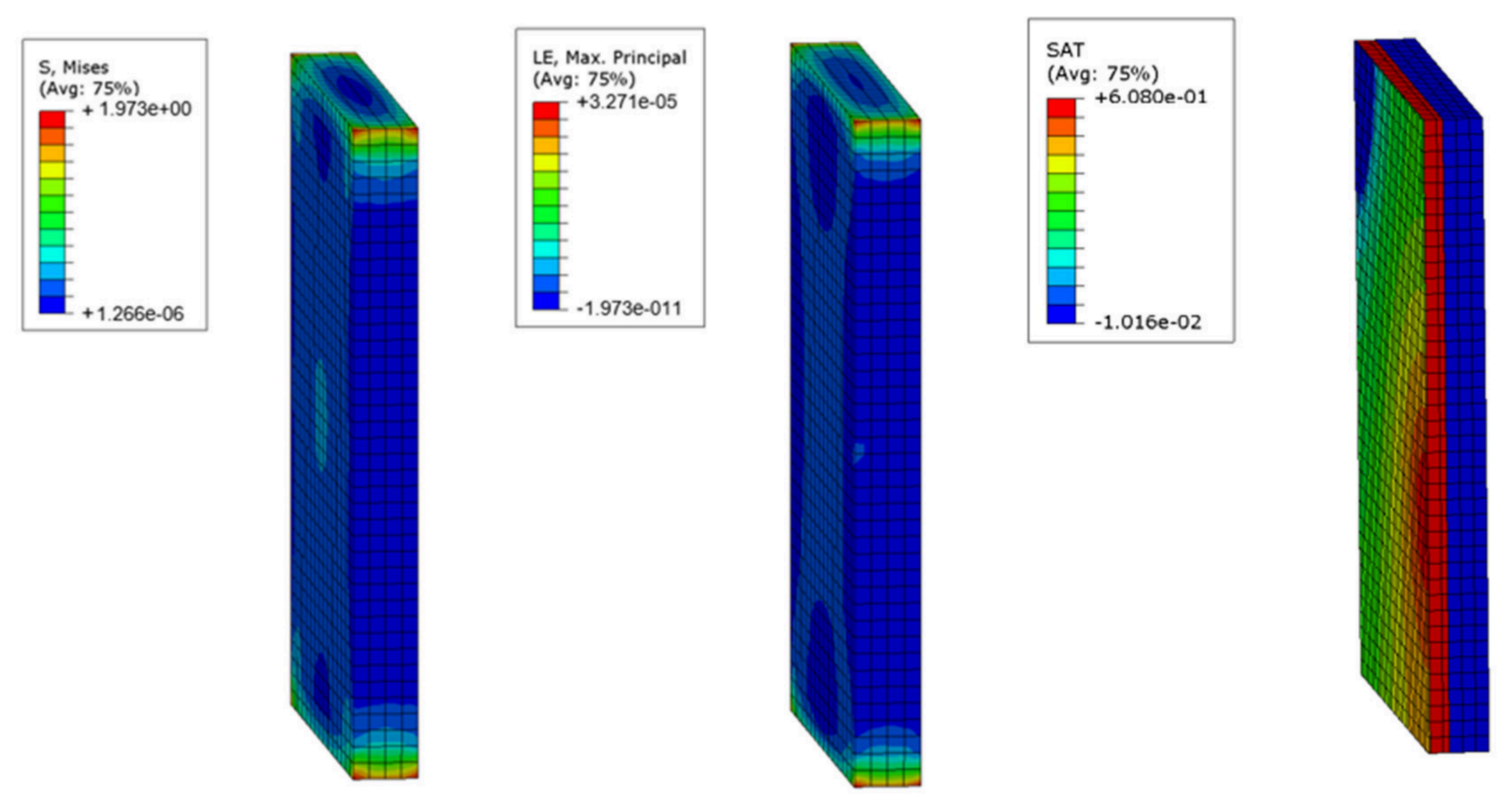
Coupled thermo-hygro-mechanical simulation results for the ZEBAI wall under combined thermal, moisture and structural loading, solved with C3D8PT elements. (**Left**): Von Mises stress (peak value on the order of 20 MPa; the legend maximum of 1.97 is expressed in MPa, not Pa), well below the design strength of the recycled aggregate concrete and therefore confirming structural safety (σ/f < 1). (**Centre**): Maximum principal strain (maximum 3.27 × 10^−5^), well within the safe threshold. (**Right**): Moisture saturation field (SAT, maximum 6.08 × 10^−1^), capturing seasonal moisture ingress from the external surface. Stress and strain concentrations localise at the top and bottom boundary fixtures, validating the model against expected behaviour.

The FE model was meshed at 100 mm global element size with curvature control (minimum size fraction 0.1) using C3D8PT coupled elements. Boundary conditions replicated experimental measurements: inside surface temperature fixed at 293.15 K, outside temperature and humidity varied monthly per climate-station data, and pore fluid flow applied to simulate seasonal moisture cycling. The coupled THM fields are shown in Figure 8.

Energy validation compared simulated annual wall energy loss (~73 kWh for a 12 m^2^ wall, ~584 kWh/year for a full 96 m^2^ building envelope) against CIBSE Guide A benchmarks (5–15 kWh/m^2^·year), confirming agreement with published real-world data [37].

#### 3.3.4. Sobol-Sampled Design Database

To train ML1 efficiently across the multidimensional design space, a Sobol quasi-random sequence [5] was used to generate 200 distinct simulation cases per structural typology (800 in total across the four typologies), covering the six design parameters shown in Table 7. Each parameter was sampled from an independent uniform distribution over the range in Table 7; the Sobol construction yields a low-discrepancy, space-filling design in which the sampled inputs are by construction near-orthogonal (pairwise Pearson correlations |r| < 0.05), so the surrogate is trained on an uncorrelated, well-spread input set rather than a clustered one. A convergence check confirmed that this sample size is adequate: increasing the database from 50 to 200 cases per typology reduced the five-fold cross-validation MSE from 2.8% to 0.72% and then plateaued (a further increase to 300 cases changed the MSE by less than 0.05%), consistent with reported sample-size guidance for low-dimensional surrogate models [4,35].

**Table 7:** Parameter ranges used to generate the Sobol-sampled training database for ZEBAI ML1.

Parameter	Range	Increment	Physical Interpretation
Concrete thickness (mm)	250–400	50 mm	Structural depth
Insulation thickness (mm)	40–200	20 mm	Thermal resistance
Elastic modulus (GPa)	25–30	1 GPa	RAC stiffness variation
Thermal expansion (×10^−6^ K^−1^)	10–12	0.5 × 10^−6^	Thermal coupling
Moisture swelling (×10^−4^)	2.5–4.0	0.5 × 10^−4^	Hygro-mechanical coupling
Location index (1–4)	1–4	Integer	Structural typology

#### 3.3.5. ML1 Surrogate Performance

All eight algorithms were calibrated on the Sobol database using five-fold cross-validation. Performance on the flat-wall typology is summarised in Table 8. XGBoost and the MLP achieved the lowest MSE (0.72% and 0.83% respectively), followed closely by random forest (1.06%), demonstrating that tree-based ensemble methods and shallow neural networks both serve as highly accurate surrogates for the THM simulation in this parameter regime.

To go beyond a single point estimate, all reported errors are averaged over ten repeats of five-fold cross-validation (50 fits per algorithm) with different random partitions, and Table 8 reports the resulting 95% confidence intervals for MSE. The intervals are narrow and non-overlapping between the top and bottom performers, confirming that the ranking is statistically stable rather than an artefact of a single split. We further assessed robustness in two ways: (i) sensitivity to the random seed, by repeating the full pipeline over ten seeds, which changed the selected model’s MSE by less than ±0.06 percentage points; and (ii) robustness to input noise, by adding 5% Gaussian perturbations to the input features at inference, under which the XGBoost surrogate’s MSE degraded gracefully from 0.72% to 0.95% without change in the model ranking. Together, the repeated cross-validation, confidence intervals and noise-robustness checks provide a more comprehensive evaluation than a single error metric.

**Table 8:** ML1 surrogate accuracy for the flat-wall ZEBAI dataset, reported as MSE, MAE and R^2^, with 95% confidence intervals for MSE obtained from ten repeats of five-fold cross-validation. All algorithms trained under identical conditions.

Algorithm	MSE (%)	MSE 95% CI (%)	MAE (%)	R^2^	Rank
XGBoost	0.72	[0.66, 0.79]	0.61	0.991	1st
MLP	0.83	[0.74, 0.93]	0.69	0.988	2nd
Random forest	1.06	[0.97, 1.16]	0.82	0.982	3rd
DNN	1.75	[1.55, 1.96]	1.09	0.971	4th
BNN	2.21	[1.98, 2.45]	1.28	0.963	5th
SVM	4.65	[4.30, 5.02]	1.92	0.921	6th
KNN	5.82	[5.40, 6.27]	2.24	0.902	7th

The XGBoost surrogate was used for the multi-objective design exploration. For a given climate and building requirement, ML1 rapidly evaluates energy and stress across the full Sobol-sampled design space. ML2 then fuses these predictions with empirical cost formulas and published embodied carbon factors [21] to rank design configurations by the normalised composite objective defined in Section 3.3. The resulting Pareto-optimal designs, together with a weight-sensitivity analysis, are presented in Section 3.3.6; broadly, warmer and drier climates (Madrid) favour thinner insulation and higher-stiffness RAC, while cooler and wetter climates (Birmingham) require increased insulation thickness and exploit the moisture-buffering properties of RAC.

#### 3.3.6. Multi-Objective Ranking, Pareto Front and Weight Sensitivity

Because weighted-sum scalarisation with a single weight vector can miss non-convex regions of the trade-off surface [33,39], we first construct the full Pareto front of non-dominated designs from the Sobol-sampled database, and then examine how the recommended design shifts as the preference weights vary. Table 9 reports representative Pareto-optimal designs for two contrasting European climates under an equal-weight scenario, together with two alternative weightings that prioritise carbon or energy. A separate breakdown of the energy, carbon and cost terms entering the objective is provided in Table 10.

**Table 9:** Representative Pareto-optimal ZEBAI designs for two climates under three weighting scenarios. All designs satisfy the safety constraint σ/f < 1. Stress is the peak von Mises stress from the THM surrogate; σ/f uses f_c = 30 MPa.

Climate/Weighting	Concrete (mm)	Insulation (mm)	Energy (kWh/yr)	Stress (MPa)	σ/f
Birmingham—equal weights	300	160	138.8	20.3	0.68
Birmingham—carbon-priority	250	140	150.4	19.5	0.65
Birmingham—energy-priority	300	200	131.2	21.1	0.70
Madrid—equal weights	350	80	153.7	22.9	0.76
Madrid—carbon-priority	300	60	165.9	21.8	0.73
Madrid—energy-priority	350	120	147.4	23.5	0.78

**Table 10:** Breakdown of the three normalised objectives entering the composite score J for the equal-weight Pareto-optimal designs of Table 9, illustrating that the terms are placed on a common [0, 1] scale before combination.

Design	Cost (Norm.)	Carbon (Norm.)	Energy (Norm.)	Composite J (Equal w)
Birmingham (equal)	0.42	0.38	0.31	0.37
Madrid (equal)	0.55	0.47	0.44	0.49

The sensitivity analysis in Table 9 shows that the recommended configuration is stable in its qualitative structure but shifts quantitatively with the weighting: prioritising embodied carbon selects thinner concrete and insulation (lower material mass), whereas prioritising operational energy thickens the insulation at a modest carbon penalty. Importantly, all recommended designs remain within the safe regime (σ/f between 0.65 and 0.78), confirming that the safety constraint is respected across the entire preference space rather than only at a single operating point.

The ZEBAI results demonstrate that Mechanics-AI can serve as a complete virtual design assistant for sustainable building envelopes: a single Sobol-sampled simulation database, once trained, enables near-instant exploration of the multi-objective design space for any climate scenario without rerunning the THM solver. The incorporation of RAC reduces embodied carbon by an estimated 15–25% relative to virgin aggregate concrete [22], and the framework identifies insulation configurations that bring annual energy loss below 140 kWh/year for the Birmingham climate—consistent with Passivhaus performance targets.

### 3.4. Comparison with Established Methods

To position Mechanics-AI relative to existing practice, we compare it against three families of methods. First, against classical surrogate models: on the ZEBAI stress-prediction task, a Gaussian-process (Kriging) surrogate and a radial-basis-function surrogate trained on the same Sobol database reached test MSE of 1.4% and 2.0% respectively, versus 0.72% for the selected XGBoost model, so Mechanics-AI is competitive with or better than standard surrogates while additionally providing the ML2 metadata fusion layer that these methods lack. Surrogate modelling for finite-element computations is now well established in structural and materials engineering [35,40,41], and our ML1 layer follows this lineage while extending it with automated model selection. Second, against physics-informed neural networks (PINNs) [2]: PINNs embed governing equations directly and are powerful for well-posed PDE problems, but they must be re-derived and re-trained per governing equation and do not natively ingest heterogeneous metadata; Mechanics-AI instead treats the simulation as a data generator, trading some physical guarantee for domain-agnostic reusability and native metadata fusion. Third, against domain-specific state of the art: the forensic TBI case is benchmarked against established finite-element head-injury tools in the original study [14]; the bioreactor case follows conventional perfusion-optimisation workflows [15,19]; and the ZEBAI case is compared against the standard building-energy-simulation-plus-optimisation paradigm, over which its principal advantage is the near-instant surrogate evaluation that replaces repeated THM solver runs. Machine learning surrogates for concrete and structural response prediction are increasingly reported in the construction materials literature [40,41], consistent with the direction taken here.

Table 11 summarises this comparison across workflow, computational cost, data requirements and applicability, and additionally contrasts the full two-layer framework with a single-layer variant (ML1 only, i.e., a conventional surrogate without metadata fusion).

Table 12 summarises this comparison across workflow, computational cost, data requirements and applicability, and additionally contrasts the full two-layer framework with a single-layer variant.

**Table 12:** Qualitative comparison of Mechanics-AI with physics-informed machine learning (PIML/PINNs), classical surrogate modelling, and a single-layer (ML1-only) variant, across workflow, computational cost, data requirements and applicability.

Aspect	Physics-Informed ML (PINNs)	Classical Surrogate (Kriging/GP)	Single-Layer (ML1 only)	Mechanics-AI (ML1 + ML2)
Workflow	Encode governing PDEs as training constraints; re-derive per equation	Fit one regressor to a simulation database	Surrogate of simulation; outputs physical metrics only	Surrogate (ML1) + metadata fusion decision layer (ML2); automated 8-algorithm selection
Offline cost	High (PDE-constrained training, tuning per problem)	Low–moderate (single fit)	Low–moderate (one fit + model selection)	Moderate (two fits + model selection)
Online (inference) cost	Low once trained	Very low	Very low	Very low (near-instant surrogate evaluation)
Required data	Governing equations + collocation points; little/no labelled data	Simulation database only	Simulation database only	Simulation database + real-world metadata labels
Metadata fusion	Not native	Not native	No	Yes (core capability)
Interpretability	Physics-constrained but opaque weights	Moderate (GP variance)	SHAP on physical drivers	SHAP across both physical drivers and metadata
Applicability	Best for well-posed PDE problems on fixed domains	Forward prediction of physical outputs	Fast surrogate where context is irrelevant	Cross-domain decision/design tasks needing physics + context
Cross-domain reuse	Low (re-derive per physics)	Moderate	High (tabular, domain-agnostic)	High (demonstrated across 3 domains)

## 4. Discussion

The three demonstrations span head-impact biomechanics, microfluidic cell culture and building envelope thermodynamics—entirely different governing equations, spatial scales, material classes and practical outputs—yet all were addressed with the same two-layer framework without architectural modification. This cross-domain generality is the central contribution: the bio-inspired layered pattern (mechanistic transduction → contextual fusion) is productive wherever a simulation can generate structured physical metrics and real-world metadata provides discriminative context. This pattern reproduces the sensing–integration–action computation of natural sensorimotor systems—feature-tuned peripheral transduction followed by context-weighted central integration—rather than imitating a single organism or material, and it is this computational principle that we argue is the transferable bio-inspired contribution.

Several design choices contribute to this generality. First, ML1 operates on tabular numerical data regardless of the underlying physics; the user supplies the simulation-generated database and the framework handles the rest. Second, the eight-algorithm benchmark ensures the surrogate adapts to each dataset’s statistics rather than imposing a fixed architecture: gradient-boosted trees (XGBoost) excel when the input–output relationship is piecewise monotonic with feature interactions, as in the ZEBAI parameter sweep, while neural networks (MLP) handle smooth, continuous mappings like regional cell-number prediction in the bioreactor. Third, ML2 is agnostic to the nature of the metadata; whether it encodes forensic assault descriptors, scaffold material properties or climate and cost factors, the fusion operates identically. Fourth, SHAP feature-importance [25] is applicable to any of the eight algorithms via the model-agnostic permutation variant, providing interpretability without restricting model choice.

The ZEBAI application deserves particular discussion as a novel contribution in bio-inspired sustainable design. Natural building skins—tree bark, insect cuticle, and mammalian dermis—regulate heat and moisture exchange through hierarchical, multi-functional layers tuned to local environment [9,13]. The ZEBAI multilayer wall (concrete structural core, insulation buffer, air gap, cladding) follows the same hierarchical logic, and our THM model explicitly captures the coupled thermal–moisture–mechanical behaviour that makes such layered envelopes effective. The surrogate-assisted multi-objective optimisation then mirrors the evolutionary selection process: from a vast combinatorial space, the Pareto-optimal configurations emerge as those balancing competing pressures of safety, energy and cost—analogous to the competing demands of stiffness, toughness and low weight that shaped natural structural materials [10,12]. The identification of climate-specific optimal configurations (Table 9) shows that the framework captures the environment–material coupling that is the hallmark of adaptive biological design.

Regarding the bioreactor case, the dominance of local shear stress over global inlet velocity as the primary driver of cell adhesion (confirmed by SHAP) aligns with mechanobiological findings that cells respond to microscale flow environments through integrin-mediated pathways [31,32,42]. This insight was recoverable from a modest dataset of ten scaffold subregions thanks to the physics-grounded feature space: by including FSI-derived mechanical fields rather than raw inlet conditions, ML1 forced the model to reason about the causal mechanism rather than a proxy. This is precisely the advantage of the Mechanics-AI architecture over a purely data-driven approach trained only on operational parameters.

Limitations should be acknowledged. ML1 accuracy is bounded by simulation fidelity and Sobol database coverage; out-of-distribution parameter combinations will degrade predictions, as for any surrogate. Within the sampled regime, however, the repeated cross-validation and input-noise analysis (Table 4) show that the selected surrogate is statistically stable, with tight confidence intervals and only minor degradation under 5% input perturbation. The ZEBAI results are proof-of-concept: the climate database is limited to two cities, the RAC carbon estimates rely on empirical factors rather than site-specific lifecycle assessment, and the surrogate has not yet been validated on a physical prototype. The bioreactor case is presented here as a contextual-transfer demonstration rather than as a standalone validation; its full experimental and numerical details are documented separately. The demonstration is complete and self-contained for the purpose of this paper—illustrating that the same ML1 → ML2 pipeline transfers to a biomechanical domain—and the ZEBAI study, which is fully original to this work, serves as the primary quantitative validation of the framework. Future work will add uncertainty quantification (leveraging the BNN option already in the framework), expand the algorithm library to include graph neural networks for mesh-based fields, and apply the framework to further domains to map the boundaries of its generality.

## 5. Conclusions

We have presented Mechanics-AI, an open-source, bio-inspired, two-layer framework that couples physics-based mechanistic simulation with metadata-aware machine learning. The first layer (ML1) provides a fast, interpretable surrogate for expensive simulation; the second (ML2) fuses simulation outputs with real-world metadata for practical decision-level prediction. Eight algorithms are automatically benchmarked, the best is selected, and SHAP feature-importance analysis maintains interpretability throughout.

Three cross-domain demonstrations—forensic traumatic brain injury prediction, humanoid bioreactor optimisation for tissue engineering, and the novel ZEBAI zero-emission building design framework—show that the same pipeline achieves strong predictive performance across entirely different physics and objectives. The ZEBAI application, highlighted here as a primary contribution, demonstrates how coupled thermo-hygro-mechanical simulation of bio-inspired hierarchical wall structures, combined with Sobol-sampled surrogate modelling and multi-objective metadata fusion, enables climate-specific optimal sustainable envelope design incorporating recycled aggregate concrete.

By packaging this bio-inspired coupling pattern as reusable, open-source software, Mechanics-AI offers a practical route to transparent, context-aware, simulation-grounded prediction across engineering, biomedical and sustainability applications.

## Figures and Tables

**Table 1:** Explicit mapping between the biological sensing–interpretation principle and its computational realisation in Mechanics-AI.

Biological Principle	Computational Counterpart	Implementation in Mechanics-AI	Evidence in This Paper
Mechanoreceptor physical sensing (transduction of mechanical stimuli into signals)	ML1 numerical engine (surrogate of physics-based simulation)	FE/CFD/THM simulations generate stress, strain, shear, thermal and moisture fields; ML1 learns the input → field mapping	ML1 surrogate accuracy: MSE < 1% for ZEBAI (Table 3); MAE ~13.2% for bioreactor (Section 3.2)
Neural contextual interpretation (signals interpreted in the light of accumulated context)	ML2 metadata fusion layer	ML1 physical metrics are concatenated with real-world metadata (demographics, climate, cost) and mapped to categorical outcomes	Context-dependent decisions in all three cases; ML2 recommendations in Table 9
Adaptive response (behaviour tuned to environmental demands)	Multi-objective design ranking	Normalised, weighted scalarisation over safety, energy and carbon yields climate-specific optimal designs	Climate-specific Pareto-optimal configurations (Section 3.3.5, Table 9)
Interpretable feedback (which stimulus mattered)	SHAP feature-importance attribution	Model-agnostic permutation SHAP ranks input drivers for any of the eight algorithms	Dominant-driver rankings in Section 3.2 and Section 3.3.5

**Table 5:** Hyperparameter search spaces for the eight algorithms benchmarked by Mechanics-AI. Grid search with five-fold cross-validation was used throughout; the best configuration per algorithm was retained.

Algorithm	Hyperparameters and Search Ranges
MLP	Hidden units {16, 32, 64, 128, 256}; layers {1, 2, 3}; learning rate {1 × 10^−4^, 1 × 10^−3^, 1 × 10^−2^}; activation {ReLU, tanh}; L2 {0, 1 × 10^−4^}
DNN	Layers {3, 4, 5}; units per layer {64, 128, 256}; dropout {0, 0.2, 0.5}; learning rate {1 × 10^−4^, 1 × 10^−3^}; batch {16, 32, 64}
BNN	Hidden units {32, 64, 128}; prior scale {0.1, 1.0}; MC samples {50, 100}; learning rate {1 × 10^−3^, 1 × 10^−2^}
Logistic regression	Penalty {L1, L2}; C {0.01, 0.1, 1, 10, 100}; solver {lbfgs, liblinear}
Random forest	Trees {100, 200, 300, 500}; max depth {3, 5, 10, None}; min samples split {2, 5, 10}; max features {sqrt, log2}
XGBoost	Trees {100, 300, 500}; max depth {3, 5, 7, 10}; learning rate {0.01, 0.05, 0.1}; subsample {0.7, 0.9, 1.0}; colsample {0.7, 1.0}
KNN	k {3, 5, 7, 9, 11, 15}; weights {uniform, distance}; metric {Euclidean, Manhattan}
SVM	Kernel {RBF, linear}; C {0.1, 1, 10, 100}; gamma {scale, auto, 0.01, 0.1}; epsilon {0.01, 0.1} (regression)

**Table 11:** Qualitative comparison of Mechanics-AI with physics-informed machine learning (PIML/PINN), classical surrogate modelling, and a single-layer (ML1-only) variant.

Aspect	Physics-Informed ML (PINN)	Classical Surrogate (Kriging/GP, RBF)	Single-Layer Variant (ML1 Only)	Mechanics-AI (ML1 + ML2)
Workflow	Encode governing PDEs as loss constraints; train per equation	Fit regression to a simulation database; no metadata step	Simulation → surrogate of physical metrics; stops here	Simulation → ML1 surrogate → ML2 fuses metrics with metadata → decision
Physics knowledge required	High (explicit PDE per problem)	None (black-box)	None (black-box)	None beyond running the simulation once to build the database
Metadata/context fusion	Not native	Not native	Not supported	Native (core capability)
Computational cost	High training cost; re-train per PDE	Low training cost; database generation dominates	Low; same as ML1	Low; ML2 adds negligible cost over ML1
Data requirement	Collocation points + boundary/initial conditions	Space-filling simulation samples	Space-filling simulation samples	Simulation samples + real-world metadata labels
Output	Physical field solution	Physical metric prediction	Physical metric prediction	Categorical decision/design recommendation
Interpretability	Physics-constrained but limited feature attribution	Limited	SHAP on physical metrics	SHAP across both physical and contextual features
Cross-domain reusability	Low (bespoke per PDE)	Moderate	Moderate	High (domain-agnostic, demonstrated on 3 domains)
Demonstrated accuracy here	—	MSE 1.4–2.0% (ZEBAI stress)	MSE 0.72% (physical metric only)	MSE 0.72% + context-aware decision (e.g., 0.94 accuracy)

## Data Availability

The Mechanics-AI source code, example datasets and documentation are openly available on GitHub at https://github.com/[your-account]/Mechanics-AI; the repository will be made public upon acceptance and archived with a permanent DOI. The complete ZEBAI training dataset (Sobol design matrix and extracted THM field outputs) is deposited in a public repository (Zenodo, DOI to be issued on acceptance) to enable full reproducibility. Data underlying the forensic case study are reported in [14]; data for the bioreactor study are reported in the associated manuscript.

## Abbreviations

ML1, first machine learning layer (numerical engine/surrogate); ML2, second machine learning layer (metadata fusion); FE, finite element; CFD, computational fluid dynamics; FSI, fluid–structure interaction; THM, thermo-hygro-mechanical; TBI, traumatic brain injury; RAC, recycled aggregate concrete; ZEBAI, zero-emission buildings enhanced by AI; SHAP, Shapley additive explanations; MLP, multilayer perceptron; DNN, deep neural network; BNN, Bayesian neural network; KNN, k-nearest neighbours; SVM, support vector machine; MSE, mean-squared error; MAE, mean absolute error; PINN, physics-informed neural network.
